# An Unusual Case of Haemophilus influenzae Associated Polyarthritis: Diagnostic and Therapeutic Challenges in Concurrent Septic and Reactive Arthritis

**DOI:** 10.7759/cureus.73194

**Published:** 2024-11-07

**Authors:** Ashrit Chohan, Maahi Qureshi, Mainul Huda, Pradeep Karunakaran Thozhuthumparambil

**Affiliations:** 1 Acute Medicine, Sandwell and West Birmingham NHS Trust, Birmingham, GBR

**Keywords:** corticosteroid therapy, inflammatory polyarthritis, reactive arthrtis, septic arthiritis, septic polyarthritis

## Abstract

Septic arthritis and reactive arthritis are both recognized as distinct causes of swollen joints; however, they can, at times, overlap as causes of acute polyarthritis. Septic arthritis is an orthopedic emergency, typically caused by bacterial infection, and requires urgent antibiotic treatment and joint drainage to prevent irreversible joint damage. In contrast, reactive arthritis is a sterile, immune-mediated arthritis that occurs following infections and is managed with anti-inflammatory treatments such as corticosteroids and non-steroidal anti-inflammatory drugs (NSAIDs).

We report the case of a 47-year-old, previously healthy male presenting with acute severe polyarthritis, including both large and small joints, fever, and flu-like symptoms. Blood cultures were positive for *Haemophilus influenzae*, leading to targeted antibiotic treatment for septicemia. However, given the rapid progression of asymmetrical polyarthralgia and systemic features, reactive arthritis was also suspected, and corticosteroids were commenced. Despite this, persistent fever and worsening joint symptoms raised concerns for septic arthritis in the left knee. Arthroscopy of the left knee revealed synovitis; however, the joint fluid culture was sterile on culture. Ultimately, polymerase chain reaction (PCR) of the fluid confirmed *Haemophilus influenzae* septic arthritis. Steroids were discontinued, and arthroscopic washout alongside targeted antibiotic therapy led to improved symptoms and inflammatory markers. However, despite gradual clinical improvement, the patient continued to have persistent polyarthralgia, raising the possibility of concurrent reactive polyarthritis alongside septic arthritis. On follow-up, rheumatology is managing chronic reactive arthritis.

This case underscores the diagnostic challenges in distinguishing septic arthritis from reactive arthritis in atypical presentations, such as *H. influenzae* infection. Concurrent arthropathies must also be considered, and no guidelines have been found to address this possibility. This raises the challenge of implementing conflicting therapies, such as corticosteroids for reactive arthritis, that could potentially worsen septic arthritis outcomes. Recognizing the potential consequence of sepsis and septic arthritis, early antibiotic therapy was initiated. Furthermore, a persistent suspicion of septic arthritis, even in the presence of features suggestive of reactive arthritis, led to diagnosis and effective treatment. Further evidence-based guidelines are needed to aid clinicians in managing two or more co-presenting arthropathies.

## Introduction

Septic arthritis and reactive arthritis are both differentials for acute arthropathies. Septic arthritis is an orthopedic emergency, often caused by bacterial infection, typically affecting large joints and requiring urgent treatment to prevent irreversible joint damage. The most common pathogens are *Staphylococcus aureus* and *Streptococcus* species. However, *Haemophilus influenzae*, especially non-typeable strains, can still cause infections in adults, despite widespread vaccination against the type B strain [1]. Without prompt treatment, septic arthritis can rapidly lead to joint destruction and long-term disability, with studies showing that irreversible damage can occur within days due to the inflammatory process and cartilage destruction [2].

Reactive arthritis, on the other hand, is an immune-mediated sterile arthritis that occurs following infections, often gastrointestinal or genitourinary, but can also be triggered by respiratory pathogens. In rare cases, an overlap can exist where an initial bacterial infection leads to a reactive arthritis-like syndrome followed by a septic joint. The immune response to an infection, often 1-4 weeks earlier, causes joint inflammation without the presence of the actual pathogen in the joint space. Symptoms commonly include asymmetric polyarthritis, typically affecting the lower limbs, along with extra-articular symptoms such as enthesitis, dactylitis, and occasionally conjunctivitis or urethritis [3]. Unlike septic arthritis, synovial fluid cultures in reactive arthritis are sterile, and diagnosis is based on clinical suspicion and exclusion of other causes. Although reactive arthritis is generally self-limiting, it can become chronic in some patients, especially those with genetic predispositions like HLA-B27 positivity. Treatment usually focuses on controlling inflammation with NSAIDs or corticosteroids [4].

Differentiating between a septic and reactive arthritic joint often relies on clinical experience, as both conditions can produce similar symptoms. Septic arthritis, however, typically presents with more acute and severe symptoms, including pronounced swelling, tenderness, erythema, and restricted range of motion. In contrast, reactive arthritis generally involves milder joint symptoms and is often accompanied by extra-articular features such as tendinitis, uveitis, and urethritis.

The diagnosis of septic arthritis involves joint aspiration, which typically reveals purulent synovial fluid with a high white cell count and positive bacterial cultures. Blood tests usually show raised inflammatory markers such as C-reactive protein (CRP) and erythrocyte sedimentation rate (ESR) [5]. Treatment requires urgent antibiotics that target the causative pathogen, as well as joint drainage or surgery to prevent permanent joint damage. On the other hand, reactive arthritis requires pain relief with nonsteroidal anti-inflammatory drugs (NSAIDs) and/or corticosteroids to reduce inflammation.

This case demonstrates the diagnostic and therapeutic challenges when septic arthritis and reactive arthritis overlap. While awaiting a confirmatory diagnosis for septic arthritis, the question arises whether to commence steroid therapy to treat reactive arthritis, especially if the patient is experiencing significant symptoms.

## Case presentation

A 47-year-old male, previously healthy and well, presented with polyarthritis, pyrexia, and flu-like symptoms, including a sore throat and myalgia. He reported swelling and pain in multiple large and small joints, notably the temporomandibular joint (TMJ), knees, ankles, and metacarpophalangeal joints (MCPs). Prior to admission, he described a short history of sore throat, pyrexia, and malaise, followed by the rapid development of severe polyarthritis. He reported joint swelling and arthralgia that restricted limb movement. He also experienced generalized weakness and severe TMJ pain, which caused difficulty in opening his mouth and chewing.

The patient denied recent travel, respiratory symptoms, rashes, or weight loss. He had no history of autoimmune or joint disorders, including rheumatoid or osteoarthritis, nor any family history of similar conditions. He did report a new sexual partner a couple of months prior but stated that he practiced safe sex and denied any symptoms of urethritis as seen in sexually transmitted infections (STIs).

On examination, his vital signs showed persistent high-grade fever and tachycardia but with normal blood pressure and oxygen saturation. He appeared distressed and clammy. The clinical examination also demonstrated multiple asymmetrical swollen, severely tender joints, mainly affecting the ankles, knees, and MCPs, with marked tenderness over the TMJ. The TMJ pain was severe, limiting his oral intake. Muscle power was reduced to 3/5 in the lower limbs and 4/5 in the upper limbs, though sensations were intact. There was no evidence of organomegaly, lymphadenopathy, or skin eruptions.

Laboratory results revealed significant abnormalities, as shown in Table 1. His infection markers were notably raised, particularly neutrophils and CRP. Procalcitonin levels were elevated, suggesting a bacterial infection. An extensive septic screen was performed to investigate the source of the infection. There were no consolidative features on the chest X-ray, and the urinalysis was negative for nitrites and leukocytes, with no growth on the urine culture. The STI screen and viral serology (HIV, hepatitis B and C, Epstein-Barr virus, and cytomegalovirus) were negative. Cerebrospinal fluid (CSF) analysis showed no abnormalities, and computed tomography (CT) scans of the neck, thorax, abdomen, and pelvis revealed no infection source. The echocardiogram showed no signs of infective endocarditis. However, blood cultures were positive for *Haemophilus influenzae*.

**Table 1:** Laboratory results on admission CRP: C-reactive protein, MCS: microscopy, culture, and sensitivity, STI: sexually transmitted infection.

Laboratory tests		Value	Reference range
Full blood count	White blood cell (x10^9 ^/ L)	16.40	(4.00-11.00)
	Red blood cell (x10^12^/ L)	4.62	(4.5-6.00)
	Haemoglobin (g/L)	151	(125-180)
	Haematocrit (L/L)	0.437	(0.4-0.5)
	Neutrophils (x10^9 ^/ L)	14.63	(1.7-7.5)
	Lymphocytes (x10^9 ^/ L)	1.26	(1.0-4.0)
	Monocytes (x10^9 ^/ L)	0.44	(0.2-0.8)
	Basophil (x10^9 ^/ L)	0.03	(0.0-0.1)
	Eosinophil (x10^9 ^/ L)	0.03	(0.1-0.4)
	Platelet (x10^9 ^/ L)	101	(150-400)
Coagulation screen	International normalized ratio (INR)	1.2	(0.8-1.2)
Urea and electrolytes	Sodium (mmol/L)	140	(135-145)
	Potassium (mmol/L)	3.7	(3.5-5.5)
	Urea (mmol/L)	4.8	(2.5-7.8)
	Estimated glomerular filtration rate (eGFR)–(mL/min)	>90	(>90)
	Creatinine (umol/L)	66	(59-104)
Inflammatory markers	CRP (mg/L)	304	<5
	Procalcitonin (ug/L)	4.43	(<0.05)
Liver function tests	Bilirubin (umol/L)	17	(<21)
	Alanine aminotransferase (U/L)	45	(45)
Miscellaneous	Blood culture	Both aerobic and anaerobic bottles showed growth of *Haemophilus influenzae*	
	Malaria parasite screening	Negative	
	Urine MCS	No growth	
	Urate level	Within normal limits	
	Autoimmune/vasculitis screening	Unremarkable	
	Complements	Mildly raise C3, Normal C4	
	Immunoglobulins	Within normal limits	
	STI screen (Chlamydia, Gonorrhoea)	Negative	

He was initially treated with broad-spectrum antibiotics, ciprofloxacin, and vancomycin (due to a penicillin allergy). Once antibiotic sensitivities were confirmed, this was changed to ceftriaxone, which was later escalated to meropenem due to persistent pyrexia and raised infection markers. A rheumatology consult was sought due to the polyarthritis and systemic features preceded by upper respiratory tract symptoms. A provisional diagnosis of reactive arthritis was made, and prednisolone was administered as per rheumatology advice. He showed marginal improvement in his symptoms while on steroids.

**Figure 1:**
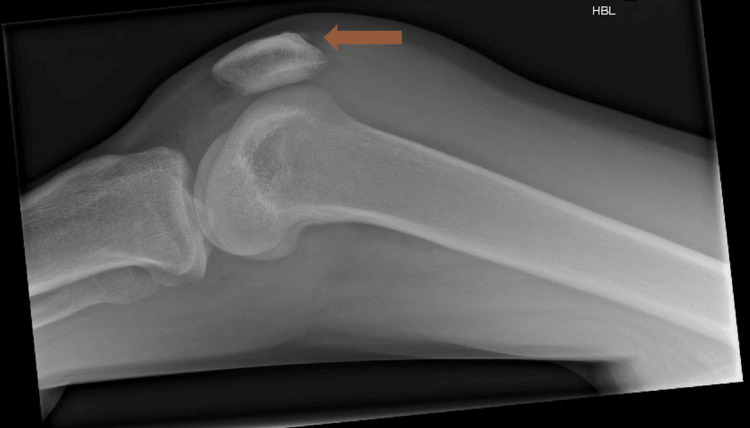
Left knee X-ray (AP, horizontal-beam lateral) demonstrating mild reduction of knee joint spaces suggesting early degenerative changes with small to moderate sized effusions in suprapatellar joint recesses (denoted by orange arrow)

Despite the initial improvement, his symptoms were persistent, and inflammatory markers continued to be elevated. This, alongside the positive blood cultures, raised concerns about septic arthritis. Prednisolone was stopped after three days because of this concern. His left knee, in particular, showed significant swelling and tenderness. X-rays of the left knee demonstrated mild joint space narrowing and small-to-moderate effusion (Figure 1). A knee arthroscopy performed by the orthopedic team identified synovitis with frothy, reddish fluid. Synovial fluid culture did not grow any bacteria; however, PCR returned positive for *Haemophilus influenzae* several days later. A diagnosis of *Haemophilus influenzae* septic arthritis was confirmed, antibiotics were switched to levofloxacin, and the patient underwent arthroscopic washout of the left knee joint. Prednisolone was given for four days in the interim period between the negative synovial culture results and positive PCR results. 

Following this, his laboratory markers improved, with CRP, white cell count, and pro-calcitonin levels normalizing. His systemic symptoms subsided, and his mobility gradually improved with therapy and analgesia. However, on follow-up with the orthopedic and rheumatology teams two months after discharge, he continued to experience polyarthralgia. He was started on a tapering course of steroid therapy, which led to further symptom improvement but incomplete resolution. In the latest rheumatology follow-up, he is currently being considered for disease-modifying antirheumatic drugs (DMARDs) for persisting/chronic reactive arthritis.

## Discussion

The primary diagnostic challenge was distinguishing between septic arthritis and reactive arthritis, especially as their management contrasts significantly. The unusual nature of the presentation was not typical of classical reactive or septic arthritis. Furthermore, *H. influenzae* was cultured in the blood, and whilst it is associated with both diagnoses, it has been rare since vaccination came into practice [6]. The literature has shown that *H. influenzae *associated septic arthritis typically manifests as monoarthritis rather than polyarthritis, though cases of polyarthropathy secondary to* H. influenzae* also exist [7, 8]. Similarly, respiratory pathogens like *H. influenzae *are less common triggers for reactive arthritis. Cases of reactive arthritis triggered by *H. influenzae* meningitis have been reported more so than respiratory infection [9, 10].

Septic arthritis requires prompt recognition and immediate treatment to prevent irreversible joint damage. In this case, while blood cultures revealed *H. influenzae*, the sterile nature of the synovial fluid and the absence of organisms on culture reasoned against septic arthritis. Reactive arthritis is less acute than septic arthritis but can still cause significant discomfort and disability. The marked improvement of symptoms with corticosteroids supported the diagnosis of an immune-mediated inflammatory process, like that of reactive arthritis. X-ray imaging of both knees showed early degenerative changes and effusions but no erosive changes typically seen in chronic inflammatory arthritis such as rheumatoid arthritis. The patient was reviewed multiple times by both the orthopedic and the rheumatology teams, with ongoing uncertainty regarding the diagnosis of septic arthritis versus reactive arthritis. The subsequent arthroscopy of the left knee confirmed synovitis, with frothy fluid, supporting an inflammatory process. Synovitis is seen in both septic and reactive arthritis, but the absence of organisms in the joint fluid favoured reactive arthritis. However, it is worth noting that antibiotics were initiated immediately on presentation and continued throughout admission and may have contributed to cultures failing to grow *H. influenzae*. The other affected joints were not suitable for joint aspiration.

PCR testing of the synovial fluid confirmed *H. influenzae*. Based on existing case reports, this could suggest a possible diagnosis of septic polyarthritis caused by *H. influenzae* [9, 10]. However, the other joints were unsuitable for aspiration, and continued antibiotic therapy may have inhibited culture growth. Furthermore, with limited evidence and the patient being immunocompetent with no significant past medical history, septic polyarthritis is unlikely. A more probable diagnosis is monoarticular septic arthritis secondary to *H. influenzae* bacteremia associated with an upper respiratory tract infection. This would follow that the other small joints, including the MCP and TMJ joints, would be due to concurrent reactive arthritis, as this can occur following *H. influenzae* infection, albeit uncommonly.

The significance of distinguishing between the two diagnoses is the contrasting management options. During early admission, broad-spectrum antibiotics (vancomycin and ciprofloxacin) were used, given the high suspicion of bacterial sepsis. After *H. influenzae* was identified, the treatment regimen was modified according to culture sensitivities whilst sourcing the infection continued. Early antibiotic treatment is essential to manage the underlying infection and may help prevent or reduce the severity of post-infectious complications like reactive arthritis [11]. The possibility and complications of septic arthritis were also being considered with the view to continue antibiotics until septic arthritis could be firmly excluded.

Despite receiving appropriate antibiotic treatment guided by microbiology advice, the patient showed minimal clinical improvement. The sterile joint fluid was convincing of reactive arthritis, and steroid therapy was recommenced. Corticosteroids are typically avoided in septic arthritis due to the theoretical risk of worsening sepsis. However, recent reviews and case reports suggest corticosteroids, especially those used concurrently with antibiotics, may improve clinical outcomes in septic arthritis, although further randomized controlled trials and studies are required before treatment recommendations are made [12, 13]. The clinical impact of steroid therapy on the patient remains unclear. Although there was an initial reduction in pain in the joints, the patient continued to experience moderate to severe arthritic symptoms, along with persistently elevated infection markers and fever. The most effective intervention proved to be a joint washout procedure performed by the orthopedic team and targeted antibiotic therapy. Only after these treatments did the patient demonstrate significant clinical and biochemical improvement, supporting the diagnosis of septic arthritis in the left knee.

As a long-term consequence of this admission, the patient continues to experience mobility challenges and has yet to regain his full functional baseline. Although his left knee has shown significant improvement, mild arthralgia persists, likely due to residual joint damage from septic arthritis. His ongoing polyarthralgia is being managed by the rheumatology team, who are evaluating him for chronic reactive arthritis and considering disease-modifying antirheumatic drug (DMARD) therapy after corticosteroids failed to completely resolve his symptoms.

## Conclusions

Considering the case presentation, investigative findings, and current literature, we propose that the pathophysiology likely involved an initial upper respiratory tract infection caused by *Haemophilus influenzae*, with the subsequent haematogenous spread leading to septic arthritis of the left knee. It may have simultaneously led to several other joints affected either by an immune-mediated reaction (reactive arthritis) or by septic spread (septic polyarthritis), with the former being the most probable explanation. Confirmation is challenging, as aspiration of additional joints was not possible, and continuous antibiotic therapy may have prevented significant growth on cultures. Nonetheless, antibiotic therapy was essential to prevent potential septic joint destruction. Corticosteroid therapy may have played a role in alleviating the symptoms of reactive arthritis during admission but theoretically may have exacerbated the septic arthritis of the left knee.

This case highlights several important learning points for clinicians. First, to maintain a high clinical suspicion of septic arthritis in cases of polyarthritis with systemic features. Early septic screening and empirical broad-spectrum antibiotic therapy are crucial to prevent the long-term sequelae of septic arthritis, even if reactive arthritis is the working diagnosis. Secondly, concurrent arthritic diagnoses may present. With no guidelines that address a dual arthritic picture, future cases like this may be difficult to navigate. As such, clinicians should adapt and tailor therapy to the clinical picture. While prompt antibiotic therapy is necessary for treating septic arthritis, the use of immunosuppressants such as corticosteroids may be required to manage the symptoms of reactive arthritis, especially once the initial septic burden is reduced. Corticosteroids are typically avoided in septic arthritis; however, they may have a future role in its treatment alongside antibiotics, as the literature suggests. Further evidence-based guidance needs to be developed to aid clinicians in managing multiple concurrent arthropathies, especially when the treatments may conflict.
